# Estrogen–ER‐α axis induces PNPLA3 p.I148M protein variant to promote steatotic liver disease susceptibility in women

**DOI:** 10.1002/ctm2.1524

**Published:** 2024-01-15

**Authors:** Alessandro Cherubini, Elia Casirati, Serena Pelusi, Luca Valenti

**Affiliations:** ^1^ Department of Transfusion Medicine Precision Medicine Lab, Biological Resource Center, Fondazione IRCCS Ca' Granda Ospedale Maggiore Policlinico Milan Italy; ^2^ Department of Pathophysiology and Transplantation Università Degli Studi di Milano Milan Italy

1

Metabolic dysfunction‐associated steatotic liver disease (MASLD) is the leading cause of liver disease and its incidence is increasing worldwide.^1^ In patients with MASLD excess accumulation of liver fat is linked to metabolic alterations such as insulin resistance, obesity and type 2 diabetes.^1^ MASLD encompasses a wide spectrum of hepatic alterations ranging from uncomplicated steatosis to severe lipotoxicity leading to metabolic dysfunction‐associated steatohepatitis (MASH), fibrosis and cirrhosis and is becoming the leading cause of hepatocellular carcinoma and then liver transplantation.^1^ MASLD is a heterogeneous condition with a strong heritable component. Notably, the patatin‐like phospholipase domain containing 3 (*PNPLA3*) p.I148M variant is the main genetic modifier of MASLD susceptibility, but the molecular mechanisms underpinning the liver phenotype expression are still debated, although it seems to require an accumulation of the mutant protein on lipid droplets in hepatocytes.^2^ Women are generally protected against MASLD by the metabolic regulation exerted by estrogens, exerting beneficial effects on lipid metabolism at the systemic level and in hepatocytes mainly through the estrogen receptor‐alpha (ER‐α). However, at menopause estrogen levels drop and protection against liver disease is lost, with a fraction of women developing rapidly progressive liver disease.^3^ Also supporting the protective role of estrogens, the incidence of MASLD in menopausal women taking hormonal replacement therapy was higher than in pre‐menopausal ones, but still lower than in menopausal women.^4^ On the other hand, in pre‐menopausal women high concentrations of free testosterone are associated with a more than twofold higher risk of MASH.^5^ In keeping, post‐menopausal women with higher testosterone were found at greater risk of MASLD.^6^ In this respect, therapeutic options taking into account androgen‐blocking drugs have shown to improve markers of hepatic fat and insulin resistance in patients with histologically proven MASLD.^7^

Previous studies have shown that adiposity and insulin resistance synergise with the PNPLA3 p.I148M variant in determining the development and progression of MASLD.^8^ However, the mechanisms explaining sex biological specificities in liver disease susceptibility are largely unknown.

To examine whether an interaction between female hormones and *PNPLA3* p.I148M variant influences MASLD progression, observations from genetic epidemiological and molecular studies were integrated in a recent study from our group.^9^ We started revealing that in menopausal women (≥55 years), who retained higher 17β‐estradiol (E2) levels than men, carriage of the p.I148M variant conferred a larger increase in the risk of development and progression of MASLD risk than in men in complementary clinical cohorts: (1) European individuals at risk of MASLD with histological evaluation of liver damage; (2) a case–control study of patients with end‐stage MASLD and controls, and (3) the population‐based UK Biobank cohort. Importantly, we demonstrated a multiplicative interaction between carriage of the variant and liver disease phenotypes, whereas other genetic risk variants for MASLD had larger impact in males.^9^

Next, transcriptomic analysis in obese individuals showed that insulin resistance, carriage of p.I148M and female sex independently correlated with higher *PNPLA3* expression, suggesting the mechanism amplifying the phenotype in women may be related to more abundant p.I148M accumulation. Indeed, women carrying the variant had upregulation of gene expression pathways related to inflammation and fibrosis.

We next checked in mice whether upregulation of Pnpla3 was also detected in females and affected by hormonal levels. Hepatic *Pnpla3* expression resulted higher in females during the follicular phase of the cycle characterised by high E2 levels than during the luteal phase and then in males, suggesting a direct role of estrogens in modulating *PNPLA3* liver expression.

To investigate the molecular mechanism underpinning estradiol‐related *PNPLA3* induction, human hepatoma HepG2 cells, homozygous for p.I148M, were treated with the ER‐α agonists tamoxifen and E2, resulting in upregulation of *PNPLA3* mRNA expression, protein synthesis and accumulation on intracellular lipid droplets, and leading to increased intracellular lipid droplet content. Moreover, treatment of primary human liver organoids with tamoxifen corroborated the role of this potent ER‐α agonist as a modulator of *PNPLA3* transcription. Interestingly, when co‐culturing HepG2 hepatocytes with LX2 immortalised human hepatic stellate cells in 3D multilineage spheroids in the presence of fatty acids to mimic MASH, exposure to tamoxifen increased the deposition of collagen, the hallmark of liver disease progression.

We next investigated whether ER‐α may directly induce PNPLA3 transcription. We identified at the *PNPLA3* promoter one estrogen receptor response element (ERE‐1) highly conserved in mammals, showing by chromatin immunoprecipitation and luciferase assays that upon exposure to agonists ER‐α binds ERE‐1 promoting PNPLA3 transcription. To corroborate these findings, we knocked‐out ERE‐1 in HepG2 cells by CRISPR‐Cas9 and proved how the loss of this short DNA motifs hampers PNPLA3 induction, accumulation of the p.I148M protein on lipid droplets, intracellular lipid accumulation and fibrosis in response to ER‐α agonists.

All in all, these results demonstrate an interaction between female sex and the *PNPLA3* p.I148M variant in determining MASLD, contributing to sex‐specific differences in the susceptibility to the most common cause of liver disease. Estrogen–ER‐α induction of the *PNPLA3* p.I148M variant protein in hepatocytes promotes fatty acid accumulation, competing with its paralogue PNPLA2, leading to lipid droplets accumulation^2^ (Figure 1). Although these findings should be confirmed in non‐European individuals, they may be used to design precision medicine approaches targeted to women or in individual with higher estrogen levels carrying *PNPLA3* p.I148M variant. Indeed, hepatic *PNPLA3* silencing in carriers of the variant is currently under evaluation in clinical trials in patients with MASH.^10^ Further in vivo prospective investigations are needed to determine the specific activity of hormones on the ER‐α/PNPLA3 axis in pre‐menopausal versus post‐menopausal women, as well as in men where MASLD progression is matched with higher estrogen levels.

**FIGURE 1:**
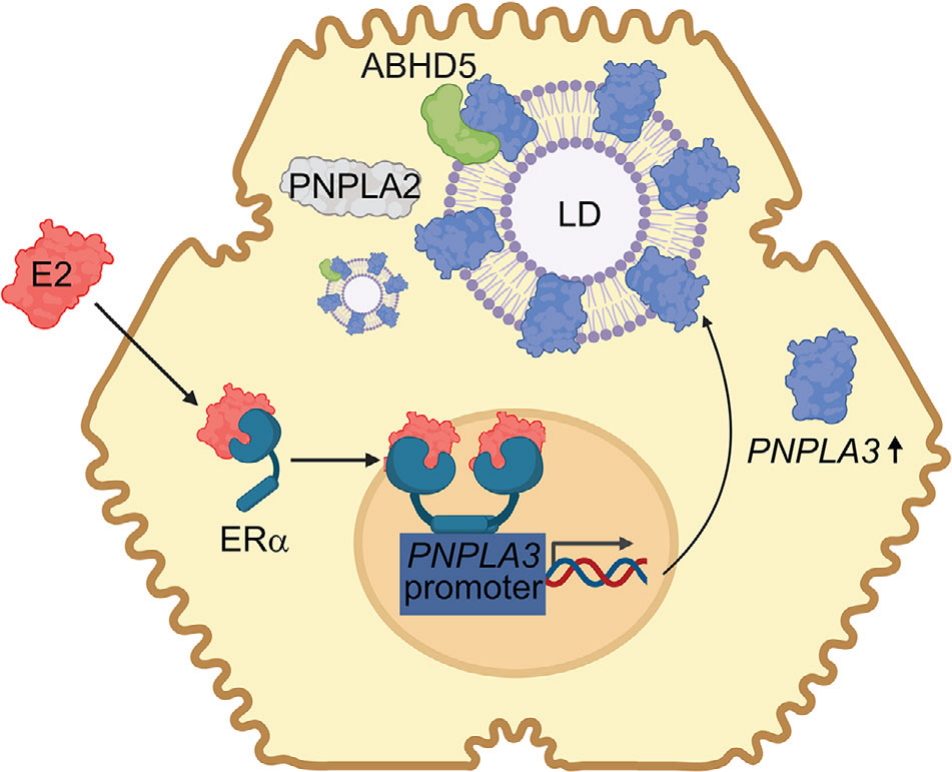
Schematic representation of sex‐dependent molecular mechanism regulating PNPLA3 p.I148M protein levels in metabolic dysfunction‐associated steatotic liver disease. Abbreviations: ABHD5, abhydrolase domain containing 5, lysophosphatidic acid acyltransferase; E2, 17β‐estradiol; ER‐α, estrogen receptor‐alpha; LD, lipid droplet; PNPLA2, patatin‐like phospholipase domain containing 2; PNPLA3, patatin‐like phospholipase domain containing 3.

Based on these findings, it will also be important to examine the effects on liver health of therapeutic or contraceptive estrogen supplementation in subjects from the general population, and of estrogen receptor modulators in women with breast cancer (e.g., tamoxifen and aromatase inhibitors) stratified by *PNPLA3* genotype.

## AUTHOR CONTRIBUTIONS

All authors have contributed equally to manuscript writing.

## CONFLICT OF INTEREST STATEMENT

Prof. Luca Valenti has received speaking fees from MSD, Gilead, AlfaSigma and AbbVie, served as a consultant for Gilead, Pfizer, AstraZeneca, Novo Nordisk, Intercept, Diatech Pharmacogenetics, Ionis Pharmaceuticals, Boeringher Ingelheim, Resalis, and received research grants from Gilead. All the remaining authors declare that they have no conflicts of interest relevant to the present study.

## ETHICAL APPROVAL

Not applicable.
